# Prevalence of Thyroid Disorder in Pregnant Women Visiting a Tertiary Care Teaching Hospital: A Descriptive Cross-sectional Study

**DOI:** 10.31729/jnma.5529

**Published:** 2021-01-31

**Authors:** Gita Khakurel, Chandrima Karki, Sanat Chalise

**Affiliations:** 1 Department of Physiology, Kathmandu Medical College and Teaching Hospital, Duwakot, Bhaktapur; 2 Department of Obstetrics and Gynaecology, Kathmandu Medical College and Teaching Hospital, Sinamangal, Kathmandu, Nepal; 3 Department of Pathology, Kathmandu Medical College and Teaching Hospital, Sinamangal, Kathmandu, Nepal

**Keywords:** *pregnancy*, *thyroid disorder*, *thyroid hormones*

## Abstract

**Introduction:**

The function of the thyroid gland is altered during pregnancy. Thyroid disorders during pregnancy are associated with serious maternal and fetal outcomes. Universal screening for thyroid disorders is recommended in the first trimester of pregnancy. This study aims to determine the prevalence of thyroid disorder during pregnancy in women attending a tertiary care hospital.

**Methods:**

A descriptive cross-sectional study was conducted in the Department of Obstetrics and Gynaecology of Kathmandu Medical College and Teaching Hospital from March 2020 to August 2020 after obtaining ethical approval from the Institutional Review committee with reference number 20032020. The pregnant women >18 years of age, irrespective of their gestational age and gravida status, were included in the study by convenience sampling method. The participants were screened by estimation of thyroid function test. Data were collected, and statistical analysis was done in Statistical Packages for Social Services version 20.0.

**Results:**

A total of 329 pregnant women were included in this study. The prevalence of thyroid disorders in the present study is 24.62%. The most common thyroid disorder observed was subclinical hypothyroidism comprising 65 (19.75%) cases followed by overt hypothyroidism 8 (2.43%) cases.

**Conclusions:**

There is a high prevalence of thyroid disorders during pregnancy in our settings. Timely screening of pregnant women helps in the early diagnosis and management of complications related to thyroid disorders.

## INTRODUCTION

Thyroid disorders are the second most common endocrine dysfunction seen in pregnancy.^1^ Various changes occur in thyroid function during pregnancy, and poor adjustments to these physiological changes result in thyroid dysfunction.^2,3^ These changes occur due to increased thyroid hormone-binding globulin (TBG) concentration, increased iodine clearance in the kidneys, and thyrotrophic effect of human chorionic gonadotropin (HCG).^4,5^

During pregnancy, optimum maternal thyroid function is essential for both the mother and the fetus.^6^ Thyroid dysfunction can have an immense impact on pregnancy outcomes and fetal development. Various adverse effects such as miscarriage, preeclampsia, anemia, low birth weight, preterm birth, increased maternal and fetal morbidity, and mortality is reported.^7^ The prevalence of hypothyroidism during pregnancy is estimated to be 0.3-0.5% for overt hypothyroidism and 2-3% for subclinical hypothyroidism.^8^

This study aims to determine the prevalence of thyroid disorder during pregnancy in women attending a tertiary care hospital.

## METHODS

This descriptive cross-sectional study was conducted among the pregnant women visiting the OPD of Obstetrics and Gynaecology Department of Kathmandu Medical College Public Limited, Sinamangal, Nepal, from March 2020 to August 2020. The study's ethical approval was taken from the Institutional Review Committee of Kathmandu Medical College Teaching Hospital, with reference number 20032020 in March 2020.

Informed written consent was obtained from each patient. History regarding the pregnant woman's age, parity, obstetric history, gestational age, past and present medical history, personal history, and family history was taken through a structured questionnaire. The pregnant women >18 years of age with a singleton pregnancy, irrespective of their gestational age and gravida status (primigravida or multigravida) were included in the study. The pregnant women with diagnosed thyroid disease, thyroid medication usage, diabetes mellitus, and hypertension were excluded from the study.

Convenient sampling was done, and sample size was calculated using the following formula:

$$\begin{array}{ll} \text{n} & ={\text{Z}}^{\text{2}}\text{p(1}-\text{p)}/{\text{e}}^{\text{2}}\\ & ={\left(1.96\right)}^{2}\times (0.31)\times (1-0.69)/{\left(0.05\right)}^{\text{2}}\\ & =329 \end{array}$$

where,

n = sample size
p = prevalence from previous study, 31%^17^.
e = margin of error (5%).
Z = 1.96 at 95% CI.

The participants were screened by estimation of thyroid function test, which includes serum free triiodothyronine (FT3), free thyroxine (FT4), and thyroid-stimulating hormone (TSH). Thyroid function tests were performed by using Maglumi Chemiluminescence Analyser. The reference ranges of the test values used in this study are as per the laboratory values used in Kathmandu Medical College and Teaching Hospital. The following normal reference ranges are recommended: TSH= 0.30 to 4.50 ulU/ml, FT3= 2-4.20 pg/ml, FT4=8.90 to 17.20 pg/ml.

According to the American Thyroid Association (ATA) guideline, pregnant women were classified into five groups^9^:

1. Subclinical hypothyroidism: High serum TSH level with normal fT4, fT3 level,
2. Overt hypothyroidism: High serum TSH level with fT4, fT3 level less than the normal range,
3. Normal
4. Subclinical hyperthyroidism: Low serum TSH level with normal fT3, fT4 level,
5. Overt hyperthyroidism: Low serum TSH level with fT3 and fT4 more than the normal range

The data was entered in SPSS (Statistical Packages for Social Services) version 20.0. The descriptive statistical analysis was done.

## RESULTS

A total of 329 pregnant women were enrolled in this study. The age range of pregnant women was 21-42 years. The maximum number of women were in the age group of 31-35 years. In the present study, 81 pregnant women out of 329 had thyroid disorders accounting for the prevalence of thyroid disorders 24.62%. The most common thyroid disorder observed was subclinical hypothyroidism comprising of 65 (19.75%) cases followed by overt hypothyroidism 8 (2.43%) cases and subclinical hyperthyroidism 6 (1.82%) cases. Overt hyperthyroidism was the least commonly seen in 2 (0.60%) cases (Table 1).

**Table 1:** Classification of thyroid disorders in pregnant women. (n = 329)

Type of thyroid disorder	Number of cases	%
Euthyroid	248	75.37
Subclinical hypothyroidism	65	19.75
Overt hypothyroidism	8	2.43
Subclinical hyperthyroidism	6	1.82
Overt hyperthyroidism	2	0.60
Total	329	100

**Figure 1:**
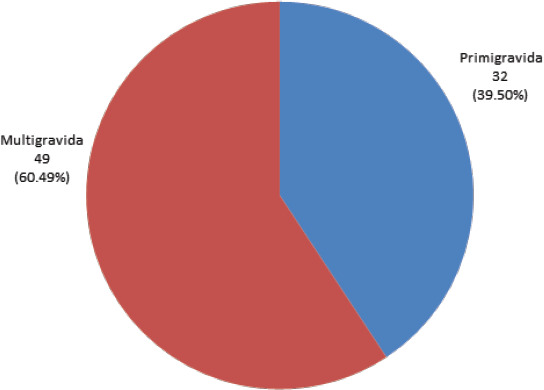
Distribution of pregnant women with thyroid disorder as per gravidity (n = 81).

**Figure 2:**
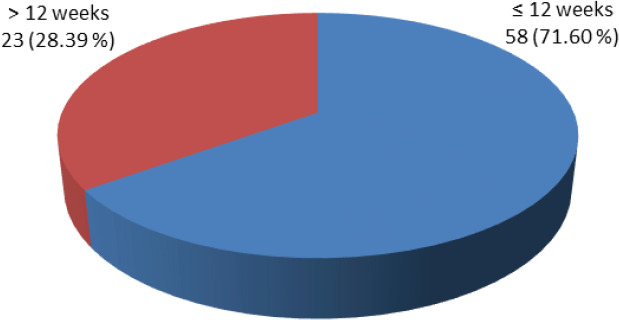
Distribution of pregnant women with thyroid disorder as per gestational age (n = 81).

Thirty-two (39.50%) pregnant women with a thyroid disorder in the study were primigravida, and 49 (60.49%) were multigravida. Among the women with thyroid disorder majority of them (71.60%) were with gestational age ≤ 12 weeks (Figure 1,2).

## DISCUSSION

Thyroid dysfunction during pregnancy has deleterious effects on both maternal and fetal outcomes.^10^ It is often missed in pregnancy due to the wide-ranging symptoms and hyper-metabolic state of pregnancy.^2^ Early identification of thyroid dysfunction and timely initiation of treatment improves the feto-maternal outcome.^11^

The prevalence of thyroid disorder in our study was 24.62%. A higher prevalence of 29% was reported in the study conducted by Shrestha B et al.^12^ in Kathmandu using trimester-specific TSH cut-off values. In contrast to this study's finding, Guan HX et al.^13^, Feki M et al.^14^ and Ajmani SN et al.^15^ observed a lower prevalence of 7.8%, 9.7%, and 13.25%, respectively. One possible reason for such variation could be the use of different upper limit cut-offs value of TSH.

In the present study, subclinical hypothyroidism was the commonest thyroid disorder seen in 65 (19.75%) pregnant women. Our finding was similar to the study done by Chaudhary LN et al.^16^ in the eastern part of Nepal, where the prevalence was 19.5%. One study from western Nepal has reported the prevalence of subclinical hypothyroidism to be 31%.^17^ In contrast to our finding, Sahu MT et al.^18^ and Ajmani SN et al.^15^ observed a lower prevalence of 6.47% and 9%, respectively.

The prevalence of overt hypothyroidism in our study was 2.43%. This finding was consistent with the study done by Saraladevi R et al.^21^, where the prevalence was 2.8%. In contrast to this study's finding, Sahu MT et al.^18^ observed a higher prevalence of 4.58%, whereas Thanuja PM et al.^19^ and Rajput R et al.^20^ reported a prevalence of 1% and 1.3%, respectively. The possible cause for hypothyroidism in the context of Nepal is insufficient intake and supply of iodine in the diet.^12^

Hyperthyroidism during pregnancy is much less common than hypothyroidism. The prevalence of hyperthyroidism is comparatively low, occurring in only 0.5-2/1000 pregnancies. Maternal hyperthyroidism is linked with obstetric complications such as preeclampsia, premature labor, low birth weight, fetal and perinatal loss.^21^ The prevalence of subclinical hyperthyroidism in our study was 1.82%, comparable to the study done by Thanuja PM et al.^19^, where a prevalence of 1.3% was reported. A higher prevalence of 3.3% and 4.2% were observed in the study done by Rajput R et al.^20^ and Taghavi M et al.^22^, respectively. However, a prevalence of 0.75% was seen in a study by Ajmani SN et al.^15^ which is less when compared to the present study.

The present study observed the prevalence of overt hyperthyroidism to be 0.6%. Similar prevalence of 0.5% and 0.4% were reported in the study conducted by Ajmani SN et al.^15^ and Rajput R et al.^20^ respectively, whereas a prevalence of 2% was observed in the study by Thanuja PM et al.^19^

Our study used a single thyroid function test to screen pregnant women regarding the limitations of our study. We did not follow up with the women. Secondly, we did not measure antithyroid antibodies, which could have given more accurate results. Thirdly, our study was a hospital-based cross-sectional study with a limited sample size. This cannot be generalized to Nepalese women until the study is done in a community with large sample size.

## CONCLUSIONS

The outcome of the study shows a high prevalence of thyroid disorder among pregnant women. Our study's findings emphasize routine monitoring of thyroid hormone in pregnant women to minimize the feto-maternal complications during pregnancy and after birth.
